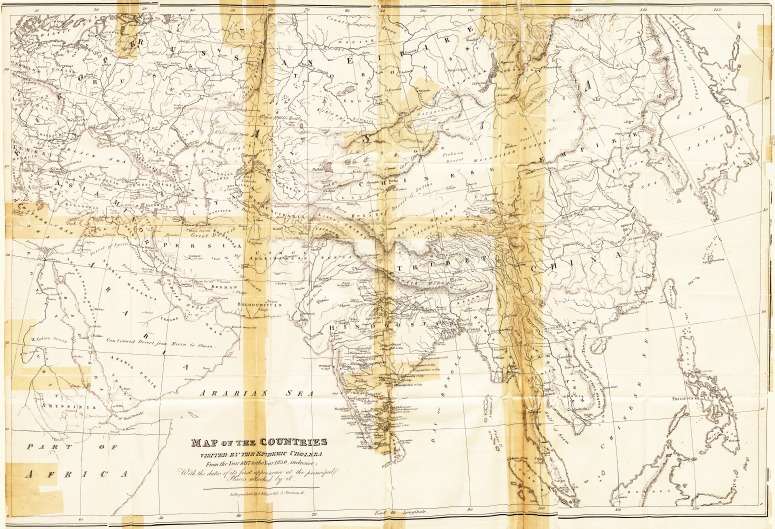# A Treatise on the Epidemic Cholera; Containing Its History, Symptoms, Autopsy, Etiology, Causes, and Treatment

**Published:** 1833-04

**Authors:** Alexander Turnbull Christie

**Affiliations:** the Honourable East-India Company's Service; Member of the Royal Asiatic Society of Great Britain and Ireland, of the Wernerian and Royal Medical Societies of Edinburgh, &c.


MAP OF THE COUNTRIES

VISITED BY THE EPIDEMIC CHOLERA

THE LONDON

### Medical and Physical Journal.

410, VOL. LXIX.] APRIL 1833. [82, New Series.

"For many fortunate discoveries in medicine, and for the detection of numerous errors, the world is indebted to the rapid circulation of Monthly Journals; and there never existed any work to which the Faculty in Europe and America, were under deeper obligations than to the 'Medical and Physical Journal of London,' now forming a long but an invaluable series."—RUSH.

### ORIGINAL PAPERS.

### THE EPIDEMIC CHOLERA.

*A Treatise on the Epidemic Cholera; containing its History, Symptoms, Autopsy, Etiology, Causes, and Treatment.* By ALEXANDER TURNBULL CHRISTIE, M.D., of the Honourable East-India Company's Service; Member of the Royal Asiatic Society of Great Britain and Ireland, of the Wernerian and Royal Medical Societies of Edinburgh, &c.

(*With a Map of the Countries visited by the Epidemic Cholera.*)

THE observations on the Cholera published by the author in 1828 form the basis of the present essay. They have been already honoured with the approbation of many distinguished physicians and journalists, both in this country and on the continent, and also by certain members of the Institute of France, at a public meeting of that learned body in November 1830. He therefore trusts that it will not be considered presumptuous in venturing to offer the present more extensive remarks for the consideration of his professional brethren. Many parts of his former essay have been completely recomposed, and very much extended; the objections offered to some parts of it have been fairly met and discussed, and advantage has been taken of all the new facts which have been subsequently published, and of the observations of some intelligent friends. His general views have been thus strongly confirmed, while a few of the less important have been slightly modified. But in addition to the contents of the treatise alluded to, the present contains so much additional matter that it must be considered as entirely new. The history of the disease, the chapters on its symptoms and on its causes are new, and large additions have been made to those on its etiology and treatment. No essential change has been made in his theory of the disease; and he is proud to say that new facts, which from time to time have become known, have only tended to develope and confirm it.

He need not repeat what he said in the preface to his former work respecting the extensive advantages he enjoyed during a five-years' residence in India for studying the disease in all its forms; for he hopes that the following pages will be found to contain sufficient evidence of them. At the same time, he wishes it to be distinctly understood that he has not confined himself exclusively to his own experience, but has sought for information in the many valuable works which have been already published by his medical brethren in India, and he is inclined to believe that he has thus been enabled to arrive at more correct views respecting the nature of the disease than could possibly have resulted from the experience, however extensive, of one individual. Although it is the very same disease which broke out in the Gangetic Delta and other parts of Bengal in 1817, which continues in India to the present day, and has spread over the greatest part of the old world, yet there can be no doubt that it has presented a variety of features at different times and places. It is therefore evident that a partial acquaintance with it would be likely to convey very erroneous notions respecting its true general character; and it will probably be found that the inaccuracies of some authors have arisen entirely from their having limited their views too exclusively to the cases which came immediately under their own observation. But in expressing himself thus, he means no disparagement to the abilities and accuracy of any of his predecessors; and it is only his good fortune to have come a little later, and thus to have it in his power to benefit by their observations, and to take a more extensive view of the disease than it was for them to command. Were a being from a distant planet to visit our globe, and confine his observations to one of the islands of the Pacific, he would certainly go away with very incorrect notions of the characters of our species; and the same would be the case were he only to pass a short time in any other single spot, and the farther he extended his journey into different regions of our little world, the more and more would he become acquainted with the nature of its inhabitants, and the errors which he at first imbibed would gradually be dissipated. So it is with every other inquiry: we have more correct notions of cholera now than prevailed upon its first appearance; and, should it continue longer in Europe, more extensive experience may remove the errors which still cling to us.

In addition to his own experience, he is indebted for much valuable information on the symptoms, autopsy, and treatment of the disease to the Reports published by order of government, under the superintendence of the Medical Boards of Calcutta,

Madras, and Bombay, and to the writings of Messrs. Annesley and Orton. Numerous works have been consulted upon the history of the disease, among which may be enumerated the Asiatic Journal, the Calcutta newspapers, the Reports already mentioned, Colonel Tod's Annals of Rajasthan, and Messrs. Baillie Fraser's travels through Persia, the Courier de St. Petersburg,* &c. He feels himself under a particular obligation to M. Alexandre Tourgeneff, by whose unwearied exertions much valuable information has been collected concerning the history of the epidemic, and its existence in Europe previous to its recent introduction from India, and which he has, in the handsomest manner, communicated without reserve.

He must add a few words respecting the map attached to this essay; for, from its necessarily imperfect state, it might, if unaccompanied by some explanation, be calculated to convey an incorrect idea in regard to the progress of the disease. Upon casting the eye over it, it may appear that the disease has generally followed the course of great rivers, or of frequented lines of communication between different countries; but this is more in appearance than reality, and is owing to various causes.

1st. In all countries the cities and large towns are situated on rivers or on great roads; and they naturally engross our attention, while the villages in the intermediate spaces are neglected; and it would not be possible, even if we had the necessary information, to insert the dates at which the disease had appeared at the small towns and villages, and thus to fill up our map.

2d. In Persia, Arabia, and Syria, we have been able to indicate the dates at which the disease first appeared, only at a few of the principal places, although it is notorious that it spread over the whole of Persia, and we may presume also over a great part of the other two countries just mentioned. Now, as the principal towns of these countries are situated on the great lines of communication between central Asia and Europe, it has been supposed by some that the disease had been carried along these roads by contagion. It would be hardly possible to represent, with perfect accuracy and in detail, its exact progress through a country, its quick passage from place to place, and its sudden appearance and disappearance; but it may be figured to the imagination as a winged restless fiend, hovering over a country, and alighting

* The author has been indebted for a perusal of this journal to the politeness of M. Smirnove.

occasionally, and in a capricious mood, to contaminate with his hateful presence the air immediately around.

Not to extend the map to an inconvenient size, it has been necessary to make the equator its southern limit, and all the places to the south of that line, which have been visited by the epidemic, have therefore been unavoidably excluded.

*History of Cholera.*

The dreadful scourge under which India and other eastern countries have been suffering for some years, and which is now extending its fatal influence into Europe, is not a new disease. It could reckon numerous victims in India, long before its fearful visitation which commenced in the year 1817, and it appears to have existed there from remote antiquity; it is said to be mentioned in the medical writings of the Hindoos, which date several hundred years before Christ, and certain forms of it were known to the Greek and Roman physicians.

Hindoostan and the neighbouring countries have always been the principal theatres of its awful ravages: they have never been without sporadic cases, have had it for years in some places as an endemic, and have been occasionally desolated by its having spread far and wide under an epidemic form.

The first well-authenticated account of its occurrence in the East is that of the Dutch physician, Bontius, who wrote in 1629, at Batavia. He describes the symptoms accurately, and refers the cause of the disease to a hot and moist disposition of the air, and to an intemperate indulgence in fruit.

We learn from the works of Colonel Tod, that it raged in Rajpootana and other parts of India, at different times, during the seventeenth century. Its first appearance is mentioned in the following passage from his annals of Mewar, where, after having described the magnificent bund or embankment of white marble, which confines the waters of the lake of Rajsmund, and which is the admiration of all travellers, he continues, "one million and one hundred and fifty thousand pounds sterling, contributed by the rana, his chiefs, and opulent subjects, were expended on this work, of which the material was from the adjacent quarries. But, magnificent, costly, and useful, as it is, it derives its chief beauty from the benevolent motive to which it owes its birth: to alleviate the miseries of a starving population, and make their employment conducive to national benefit, during one of those awful visitations of providence, famine and pestilence, with which these states are sometimes afflicted."

It was in 1661, only seven years after the accession of Raj Sing, that these combined evils reached Mewar, less subject to them, owing to its natural advantages, than any other state in India. "From all I could learn," he adds in a note, "it was the identical pestilence which has been ravaging India for the last ten years, erroneously called Cholera Morbus."

In the year 1737, or A. D. 1681, when the Rajpoots of Marwar were defending their independence against Aurungzeba, the Rajpoot chronicler thus concludes the events of that year, and incidentally introduces the existence of this pestilence. "Thus the *saca* (destruction) of Sojut was when 37 ended and 38 commenced, when the sword and the *murrie* united to clear the land." "Murrie," says Colonel Tod, "is the name for that awful scourge, the cholera morbus, which has been raging, to the loss of so many lives, for the last thirteen years, throughout India. It appears to have visited India often, of which we have given a frightful record in the annals of Mewar, in the reign of Rana Raj Sing, (see vol. i. p. 390,) in 1717, A. D. 1661, twenty years previous to this; and Orme describes it as raging in the Deckan in A. D. 1684; no doubt a continuance of the same scourge."*

Mr. Scot says,† that there is an account, in the records of the Madras Medical Board, of the disease having occurred as an endemic, in various parts of the Madras territories, in the years 1769, 1770, 1781, and 1783; and that it also occurred, in the latter year, as an epidemic all along the Coromandel coast. It was about this time that Sonnerat travelled in India, (viz. between 1774 and 1781,) and he accordingly gives an accurate description of the disease, which he denominated flux. He says, that it twice prevailed as an epidemic; that, during the first time, above 60,000 people, between Cherigam and Pondicherry, perished; and that its second visit was still more dreadful. He attributes its causes to sleeping in the open air, to eating cold rice and curds, (which is a common article of food in that country,) and to eating after cold bathing.

It is mentioned, on the authority of a committee of medical officers, who assembled, in 1819, in the Mauritias, to examine into the nature of the epidemic which then raged there, that a similar disease had occurred in that island in the year 1775.‡

In 1781, the cholera prevailed in the northern circars,

* Colonel Tod's MSS.

† Preface to the Madras Report on the Epidemic Cholera.

‡ Ibid.

and assailed, with inconceivable fury, a detachment of 5000 men, who were then marching through that part of the country, under the command of Colonel Pearse. As usually happens on such occasions, it first attacked the camp-followers, then the sepoys, and lastly the Europeans. But few officers were affected, and of these only one died. "The troops had been marching almost incessantly for six days, through sand and salt water, and were at length so enfeebled as scarcely to be able to move. A violent wind blew day and night along the whole shore, and, although it was not quite so strong at night, it was then accompanied with such a penetrating moisture as to wet through the thickest woollen clothes. The troops were, besides, in no condition to withstand the inclemency of the season: they had no tents, and few possessed even a blanket to shelter them on getting to their ground; they generally marched in the night; and many suffered by incautiously lying down while warm from exercise, and falling asleep, exposed to the influence of a damp and noxious atmosphere."*

About this period the disease spread to Calcutta, where it principally attacked the native inhabitants; and it appeared in the northern circars a second time, in 1790.

From 1781 up to 1790, it had occurred every year, and sometimes with great violence, in various parts of the Madras territories; and tolerably accurate descriptions of it, by Drs. Duffin and Davis, are to be found in the records of the Madras Medical Board. But it is to be regretted that, at this time, no post-mortem examinations were made of the fatal cases, except in two or three instances; and in these the morbid appearances are but imperfectly described.

We have no account of the cholera having occurred as an epidemic, in the Bengal provinces, before 1817; but sporadic and endemic cases had not been unfrequent. Mr. Jamieson mentions a remarkable instance of its occurrence at Hurdwar, in 1783, when an immense crowd of pilgrims, amounting, it is believed, to one or two millions, were assembled there for the purpose of ablution in the holy stream of the Ganges. The temperament at this place is very variable. The days are hot and the nights cold, with very heavy dews and chilling blasts, and the devotees were exposed to all the inclemencies of the weather. The disease broke out soon after the commencement of the ceremonies, and in less than eight days is said to have cut off more than 20,000 victims: but so confined was its influence, that it did not reach the

* Bengal Report.

village of Juwalapore, only seven miles distant, and it ceased immediately upon the concourse breaking up, on the last day of the festival."*

In the year 1817, it broke out almost simultaneously in various parts of Bengal; and this was the first time it had visited that country as an epidemic. It occurred in an unusual degree at Buddea, and other southern districts, in May and June. But it was in August that it first began to excite universal alarm by its wide spread, and the rapidity of its progress. In that month it made dreadful havoc in Jessore, a populous town situated in the centre of the Gangetic Delta, about a hundred miles north-east of Calcutta, where it was reported to have cut off, within the space of a few weeks, more than 6000 of the inhabitants. At Calcutta, "several cases of cholera occurred amongst Europeans on the 5th of September, and from that day forward the disease became daily more frequent." It commenced about the same time in numerous other parts of Bengal, and soon after the middle of September, being now strictly epidemical, it extended in all directions; "within the space of a few weeks stretching from the easterly parts of Poorneah, Dinagepoor, and Sylhet, to the extreme borders of Balasore and Cuttack, and reaching from the mouths of the Ganges nearly as high as its junction with the Jumna."

"These facts," says Mr. Jamieson, "are more than sufficient to show the fallacy of every theory which attempts to derive the disease from any local source, or to trace it to any one particular spot, as the centre from which it was emitted to the surrounding countries. They prove, without the possibility of dispute, that it broke out at very remote places at one and the same time, or at the distance of such short intervals as to establish the impossibility of the pestilential virus being, in this stage of the progress, propagated by contagion, or any of the other known modes of successive production; and that its general diffusion was therefore referrible to some cause of more universal operation.

At this period, within an area of several thousand miles, scarcely a town or village escaped; and so great was the mortality throughout the Delta of the Ganges, that the bulk of the whole population was sensibly diminished. "It is remarkable, that the large and populous city of Moorshedabad, from extent and local position, apparently very favourably circumstanced for the attacks of the epidemic, should have escaped with comparatively little loss, whilst all around was so severely scourged.

* Bengal Report, preface, p. x.

The only spots on the eastern side of the Ganges, beyond the precincts of Bengal, invaded by the epidemic, in 1817, were Moozufferpore and Chupruh, the principal stations of the Tirhoot and Sarun districts, and the cantonment of Gazeepore; and in each of these places its attacks were confined to the towns themselves, or the villages in their immediate vicinity; the great bulk of the adjoining country, at this period, having entirely escaped.

It now began to exhibit one of its most striking peculiarities: instead of bursting forth irregularly over the country, as it had hitherto done, it began to run forward in certain paths, and to confine itself within certain boundaries, which could be defined with perfect accuracy.

In the first week of November, "it reached the centre division of the grand army, then encamped, under the personal command of the Marquis of Hastings, near the banks of the Sinde, in Bundelkund."

"It was here that the disease put forth all its strength, and assumed its most deadly and appalling form. It is uncertain whether it made its first approaches on the 6th, the 7th, or the 8th, of the month. After creeping about, however, in its wonted insidious manner, for several days, among the lower classes of the camp-followers, it, as it were, in an instant gained fresh vigour, and at once burst forth with irresistible violence in every direction. Unsubjected to the laws of contact and proximity of situation, which had been observed to mark and retard the course of other pestilences, it surpassed the plague in the width of its range, and outstripped the most fatal diseases hitherto known, in the destructive rapidity of its progress. Previously to the 14th, it had overspread every part of the camp, sparing neither sex nor age in the undistinguishing virulence of its attacks: the old and the young, the European and the native, fighting men and camp-followers, were alike subject to its visits, and all equally sunk in a few hours under its most powerful grasp. From the 14th to the 20th or 22d, the mortality had become so general as to depress the stoutest spirits: the sick were already so numerous, and still pouring in so quickly from every quarter, that the medical men were no longer able to administer to their necessities. The whole camp then put on the appearance of an hospital: the noise and bustle almost inseparable from the intercourse of large bodies of people had nearly subsided; nothing was to be seen but individuals anxiously hurrying from one division of the camp to another, to inquire after the fate of their dead or dying companions, and melancholy groups of natives bearing biers of their departed relatives to the river. At length even this consolation was denied them; for the mortality latterly became so great, that there was neither time nor hands to carry off the bodies, which were then thrown into the neighbouring ravines, or hastily committed to the earth, on the spots on which they had expired, and even round the walls of the officers' tents. All business had given way to solicitude for the suffering. Not a smile could be discerned, nor a sound heard, except the groans of the dying and the wailing over the dead. Throughout the night, especially, a gloomy silence, interrupted only by the well-known dreadful sounds of poor wretches labouring under the distinguishing symptoms of the disease, universally prevailed. The natives, thinking that their only safety lay in flight, had now began to desert in great numbers; and the highways and fields, for many miles round, were strewed with the bodies of those who had left the camp with the disease upon them, and speedily sunk under its exhausting effects. It was clear that such a frightful state of things could not last long; and that, unless some immediate check were given to the disorder, it must soon depopulate the camp: it was therefore wisely determined, by the commander-in-chief, to move in search of a healthier soil and of purer air. The division accordingly, on the 13th, marched in a south-easterly direction, towards Zalgoug and Lileia, and, after several intermediate halts, on the 19th crossed the clear stream of the Betwah, and, upon its high and dry banks at Erich, soon got rid of the pestilence, and met with returning health. But its line of march, during the whole of this progressive movement, exhibited a most deplorable spectacle, although every means had been taken, by giving up the ammunition carts, and collecting elephants and draught-cattle to procure sufficient carriage, the sick were found too numerous to be moved, and were, in part, necessarily left behind; and as many who left the carts, pressed by the sudden calls of the disease, were unable to rise again, and hundreds dropt down during every subsequent day's advance, and covered the roads with dead and dying, the ground of encampment and line of march presented the appearance of a field of battle, and of the track of an army retreating under every circumstance of discomfiture and distress. The exact amount of mortality during these few calamitous days could not, from the circumstances of confusion and general disorder under which it took place, be ascertained with any degree of accuracy: from the military returns, however, it appears that, in this fatal week, of 11,500 fighting men of all descriptions, 764 fell victims to the disorder; and of the camp-followers, it was conjectured that about 8000, or one tenth of the whole, was cut off."

In the year 1818, the epidemic, still avoiding the eastern and northern parts of the country, took a south-westerly direction, and, after following the course of the principal rivers and great roads, to almost every village and town of Bundelkund and Sangor, was successively communicated to the districts of Malivah, Berar, and Kandeish, and thus extended into the presidencies of Madras and Bombay. Kotah and Jeypoor were, at this period, the most westerly places it attained; where, having encountered a high and mountainous tract, which has generally been found to be inimical to its existence, it gradually subsided, and did not penetrate across the hills, either to Ondeypoor or Ajmeer, until the year following.

The inhabitants of Ondeypoor congratulated themselves upon having so long been exempt from the epidemic, and vainly hoped that their eastern mountains would continue to oppose a sufficient barrier against its introduction; but, in July 1819, they were one day astonished and dismayed at the intelligence of their prince having been attacked in the centre of his palace, and, in a few hours afterwards, that his prime minister had fallen a victim to this cruel disease. Lieutenant-Colonel Tod, late resident at Ondeypoor, waited upon the prince during his illness, and was accompanied for a time by the minister. The prince recovered in the course of twelve or fifteen hours, in consequence, it would appear, of the exhibition of large quantities of onion-juice; a favorite Hindoo remedy in this complaint. Inquiry was then made for the minister, who had not appeared for some hours, when the melancholy intelligence was brought of his having been already carried off by the dreadful distemper from which his master had so happily escaped. It now, for the first time, made its appearance in the city, and committed sad ravages among the inhabitants.

A curious fact is recorded in regard to its course from Hoshingabad, on the river Nerbudda, to Nagpore. Many of the intermediate places were severely scourged by it, and a small town, named Mooltay, which is about half way, lost five hundred of its inhabitants; but, so very capricious was it in its visitations, that all the places between Mooltay and Negpore, and, among the rest, the large town of Baitool, were entirely exempt.

We will now trace the disease from Allahabad, at the junction of the Ganges and Jumna, to the northern provinces. It first appeared in Allahabad in March, and prevailed for several months with great malignancy, having swept off nearly 10,000 inhabitants. "The troops stationed in the port and city were not affected until the middle of July following, although holding daily and unrestrained intercourse with the townspeople." Keeping close to the banks of the Ganges, it entered Cawnpore on the 8th of April, attacked the city and neighbourhood of Beethoor; and, although it spared Bareilly, Mooradabad, and almost every other town on the same line, it broke out in Shajehanpoor in July, and is reported to have killed upwards of 5000 of the inhabitants.

It extended up the Jumna in the same way, desolating some places, while it spared others. It is remarkable that Agra was not invaded by it till the 1st of July, while Mutra, situated much higher up the river, received it so early as the beginning of June. The former is a dry, airy town, and suffered comparatively little; the latter a filthy crowded city, and had the disease in all its virulence. It commenced in Delhi on the 20th July, and continued nearly a month, making great havoc among the dense population of that large capital. It raged in the city of Meerut in July and August, and in Saharanpore in September and October; but all the intermediate places escaped. Beyond this, the epidemic could not be traced; the mountains to the north appearing to have put a stop to its further progress. But, although such has been generally observed to be the case, it has not been without exceptions, for "in June 1818 it passed the lofty range of mountains guarding Nepaul to the east, and visited Rhatmandoo, Patun, and Bhatgoon, in the subjacent valley; and, in October following, it got from Sylhet to the independent countries of Kashar and Munnipoor, on the eastern borders of Bengal: but, even here, it might be seen that the highlands were not congenial to it, for it had been raging with very great violence in the adjoining district of Sylhet, before it was enabled to overcome the obstacles opposed to its progress by the intervening mountains."*

The following extract from a letter of the Medical Board of Bengal to that of Bombay, illustrates in a striking manner how very irregular and capricious, in its visitations, the epidemic had been, at this period, in the Bengal provinces. "The disease would sometimes take a complete circle round a village, and, leaving it untouched, pass on, as if it were about wholly to depart from the district. Then, after a lapse of

* Although it has passed over many ranges of hills, I know of no instance of its having appeared at great altitudes: the Neilgerry hills, in southern India, which rise to between eight and nine thousand feet above the level of the sea, have hitherto been free from it.

some weeks, or even months, it would suddenly return, and, scarcely reappearing in the parts which had already undergone its ravages, would nearly depopulate the spot that had so lately congratulated itself on its escape. Sometimes, after running a long course on one side of the Ganges, it would, as if arrested by some unknown agent, at once stop; and, taking a rapid sweep across the river, lay all waste on the opposite bank. It rarely, however, failed to return to the tract which it had previously left. After leaving a district or town, it sometimes revisited it; but, in such cases, the second attacks were milder, and more readily subdued by medicine, than those in the primary visitation."*

Having thus given a rapid sketch of the progress of the epidemic in the years 1817 and 1818, through the provinces of Bengal, I will now trace it through the presidencies of Madras and Bombay, to the southern extremity of India; and, in doing this, it will be convenient first to follow its course along the eastern, or Coromandel coast, and afterwards through the central and western parts of the peninsula. It extended from Bengal into the Gaujam district in March 1818; from thence it gradually crept along the coast, visiting almost every town and village on its way, and reached Madras in October. It raged there from the 5th to the 24th of that month, when it received a temporary check from a violent storm which occurred on that day. It recommenced, however, but again began to decline in the beginning of November, and gradually disappeared. It had been principally confined to the lower classes. Still continuing its course to the south, it reached Cuddalore on the 14th November, Combaconum on the 20th, and Vegapatam on the 22d of the same month; it appeared at Madura on the 30th November, and it had arrived at Palamcottah, near the southern point of India, on the 1st January, 1819.

Let us now follow it through the central parts of the country. It was communicated to Nagpore, in the way we have already described, in May 1818; it appeared at Jaulnah on the 3d of July, and in the same month spread from thence N. W. to two detachments of troops in Kandeish, one of them stationed at Mulligaum, the other at Mussurabad.

It made its appearance at Punderpore on the 14th July, where crowds of strangers were congregated for the celebration of a festival, and, as usual under such circumstances, it committed great havoc. Still proceeding southward, it attacked the troops in the southern Mahratea country, and ap-

* Bombay Report.

peared in the towns of Badamee and Darwar in August. It manifested itself at Bellary on the 8th September, at Hurryheir and Chittledroog about the middle of the same month, and at the large military station of Bangalore in the end of October. This place is situated on a high table-land, having an elevation of about 3000 feet above the level of the sea; it consequently enjoys a pleasant temperature, is remarkable for its general salubrity, and never suffered much from the epidemic. In the towns of Mysore and Seringapatam, on the contrary, which are situated in a low unhealthy district, the mortality was very great, the disease having appeared there in the beginning of September. From thence it was communicated to the districts of Wynand and Coimbatore, in October and November.

From Joulnah we find that the disease had sent forth another stream, which, proceeding through Hydrabad where it arrived in the end of July, from thence to Kernal and Gooty, where it appeared on the 6th of October, it thus continued onwards, by way of Cuddapah, into the Carnatic.

Besides the towns in this part of India, already mentioned as having been visited by the disease, many others (and indeed scarcely a town escaped) were attacked by it, in the months of October and November of this year; but in no instance could it be referred to infection or contagion, many places having been attacked simultaneously, and some of the more remote places first.

We must now turn our attention to the western side of the peninsula. After having spread from Joulnah, in the beginning of July 1818, to Aurungabad and Amednuggur, the epidemic reached Seroor on the 18th or 19th, and, in the end of the same month, appeared in the city of Poonah. "On the 6th August it broke out at Panwell, a considerable village on the main line of communication between Poonah and Bombay, separated from the latter by an arm of the sea, and distant about fifteen or twenty miles; but between which a pretty constant communication is kept up by means of boats."*

On the 9th or 10th of the same month, the first case appeared on the island of Bombay. It was supposed by Dr. Taylor, of the Bombay service, to have been introduced by a man from Panwell, where he had caught the disease; but of this there is not sufficient evidence, and we must, therefore, suppose that the epidemic influence spread to this place in the same way it had done over other parts of India. From

* Bombay Report.

hence it extended along the coast to the north, and showed itself in Surat in the end of August; but of its farther progress in this direction, no distinct account has been published.

The dates of its appearance at the different towns in the Malabar coast show that it could not have been communicated from one to another; for it appeared almost simultaneously in distant places, and in some of the more southerly, at a much earlier period than in those farther to the north. Thus, it broke out in Mangalore on the 1st of September, at Cannanore on the 5th December, at Tellicherry (which is a little farther south) on the 25th November, at Calicut (still farther to the south) on the 16th of October, at Cochin on the 8th December, at Allepy and Quilon in October, and at Trivandrum in January 1819, whence it spread as far south as Cape Comorin.

Before leaving this part of the history of the epidemic, it will be necessary to add a few general remarks, in order to supply the want of more minute details, which have been unavoidably omitted in this short sketch. The disease occurred at all seasons, and in all kinds of weather; but changeable damp weather was generally observed to be favorable to its production, and its progress was sometimes checked by the occurrence of a thunder storm, or by the return of a steady temperature and dry air. Some places were observed to be much more obnoxious to its attacks than others, and high and dry situations were, every where, more exempt than low damp and insalubrious places. Ever since the first appearance of the epidemic, certain districts have been so subject to it, that few detachments of troops have passed through them without suffering. This is particularly the case with some parts of the ceded districts, of which the burial ground of Gooty bears many a sad proof; for there repose the ashes of Sir Thomas Munro, and of many other British officers who fell sacrifices to this dire scourge. The same is said of the Surat district, on the Bombay side of India.

The disease varied much in its duration and malignancy in different places. In some it raged with great fury for a few weeks or only for a few days, and suddenly disappeared; in others it continued with little or no intermission, but in a milder form, for several months; but in general it was observed to continue only for a short time, gradually increasing in intensity, to decline slowly, and to disappear within fifteen or twenty days; and few visitations continued longer than a month in one place. It sometimes returned within a very short period after it had completely subsided, or hovered about a spot, as it were, slightly changing its position, or transferring itself from one description of persons to another. The inhabitants of Gooty, for instance, suffered severely from it in 1827, while the regiment which was stationed there, and furnished guards to different parts in the town, enjoyed perfect health. On a sudden, however, the case was reversed, the townspeople were relieved from the disease, and the soldiers were attacked.

It spared neither age, sex, nor rank, but it was generally observed that the debilitated, and those exposed to fatigue and to the inclemencies of the weather, were most subject to it. Mr. Scot says, "all accounts agree in stating that the young, the healthy, and the robust, are the least liable to cholera. The observations of a great proportion of our medical officers being confined to their practice in military hospitals, we have not sufficient data to determine whether there be any peculiar liability to cholera in one sex more than in another; but, if the preceding remark be well founded, it might be inferred that the greater delicacy of females, and perhaps their greater tendency to nervous disorders, would give rise to a greater predisposition to it in them than in males.* Children are subject to cholera, but it has been observed, particularly in Mr. England's reports, that infants, who have been confined exclusively to the breast, are not susceptible of the disease. This remark, however, is to be received with reserve; as the paucity of that class of subjects, in comparison with any other, must obviously diminish the facility of forming a just conclusion." One attack gave an individual no security against a second, but appeared rather to make him more liable to it. We have no data by which we can ascertain the number of victims swept off during the first years of the epidemic in India.

In regard to the mortality of the disease in Bengal, Mr. Jameson, from extensive inquiries, comes to the following conclusions. "1st. That the sum total of the mortality occasioned by the epidemic fell far short of the rate assigned to it by the voice of the public, during the season of alarm. 2d. That the mortality was proportionately much greater among large and dense, than among small and dispersed, bodies of men. 3d. That, in a given place, it was generally greater in the commencement and middle, than towards the termination of the disorder. 4th. That, when unlimited by the intervention of remedial means, it generally amounted to

* We shall find afterwards, that in Russia this was not the case, but, on the contrary, that men were more subject to the disease than women.

one half, and sometimes to two thirds of the seizures.* 5th. That, when medical aid was daily exhibited, it rarely amounted to one third, and was generally as low as one fifth of the attacked. 6th. That men were generally more susceptible than women, and that infants and children were nearly exempt."

The following table and remarks, from the Report published under the superintendence of the Madras Medical Board, by Mr. Scot, show that in the Madras army the number of fatal cases, among Europeans, amounted to about nineteen per cent. of those attacked, among the natives of India to about twenty-three and a half per cent.

"Epidemic cholera having now existed in these territories for about five years, and, as the preceding narrative evinces, having proved a formidable scourge to all conditions of people, it will be satisfactory here to exhibit that the extent of its ravages in the army falls short of what might be apprehended from a cursory perusal of its history. The following tabular view is taken from the returns in the appendix, and it will not pass unobserved, that, prior to the appearance of the epidemic, cases of cholera were progressively becoming more frequent.

*Tabular View of the number of Cases of Cholera occurring in the Army of Fort St. George, from* 1815 *to* 1822.

| | EUROPEANS. | | NATIVES. | | STRENGTH. | |
|---|---|---|---|---|---|---|
| Years. | Admitted. | Dead. | Admitted. | Dead. | Europeans. | Natives. |
| 1815 | 65 | 0 | 87 | 0 | 13,409 | 59,672 |
| 1816 | 97 | 0 | 92 | 0 | 13,943 | 61,969 |
| 1817 | 168 | 0 | 114 | 0 | 12,959 | 61,641 |
| 1818 | 1087 | 232 | 3,314 | 664 | 10,652 | 58,764 |
| 1819 | 564 | 85 | 3,779 | 734 | 10,125 | 63,782 |
| 1820 | 356 | 69 | 3,322 | 758 | 9,416 | 76,870 |
| 1821 | 357 | 39 | 2,527 | 830 | 9,553 | 82,046 |
| 1822 | 774 | 170 | 548 | 199 | 10,813 | 74,707 |
| From 1818 to 1822, | 3,138 | 595 | 13,490 | 3,185 | | |
| Add . . | 526 | 100 | 2,340 | 550, not in the regular returns. | | |
| Total . | 3,664 | 695 | 15,830 | 3,735 | | |

The General Returns for 1815 to 1817, do not exhibit the diseases from which casualties arose, and it is not known, accordingly, whether any, or how many, of the cases of cholera, during these years, terminated in death: but in the course of the first four months of 1818, when seventeen cases of cholera took place amongst Europeans, no death ensued;

* I am rather inclined to think that this is somewhat underrated, for the disease has been certainly more fatal than this in the south of India, and also in other countries, when left to take its course.

in May, fourteen cases occurred, and nine died. Amongst the natives, during January and February, ten cases took place, without a casualty: in March, twelve cases and two deaths; in April, thirty-seven cases and thirteen deaths; in May, seventy-two cases and twenty-four deaths. We may, therefore, conclude, that the epidemic cholera furnished the first casualties amongst Europeans in May, and amongst the natives in March 1818; and that, prior to that period, the casualties from cholera, commonly called cholera morbus, did not exceed the usual proportions.

"From 1818 to 1822 inclusive, the medical returns show 3138 cases of cholera in Europeans, of which, 595 terminated fatally, being in proportion of about nineteen per centum; and 13,499 cases in natives, of which, 3185 terminated fatally, being about twenty-three and a half per centum. As, however, the medical returns of small detachments of Europeans are not always included in the general returns, and as there are no returns at all from some of these detachments, 526 cases, and their proportional one hundred casualties, are allowed, to meet the aggregate of such incidents, which then gives 3664 cases, and 695 deaths. The medical returns of various native troops being missing for certain months, recourse was had to the regimental records, from which it appears, that very nearly 550 men have died of cholera without appearing in the tables in the appendix; which number, at twenty-three and a half per centum, gives 2340 cases: with these additions, the total number of cases in the natives of the army may be stated at 15,830, and the casualties at 3,735. This loss will probably fall much within the calculations of those who have been accustomed to hear of the ravages committed by the disease.

"Great as the proportionate mortality which has just been stated may appear to be, it is nevertheless probably far within the truth. When the disease first appeared, there were many causes tending to magnify the number of attacks, and the number of cures; and a most erroneous estimate was too generally formed of the relations in which these events actually stood to each other; the regimental practitioner was accordingly astonished and dismayed at finding, when the disease attacked his corps, and each case was authenticated under his own observation, that the proportion of deaths was most widely different, and greatly exceeded his calculations.

"It is probable that, since cholera has been prevalent, many cases have been ranked under that head in the returns, which at other times, and under a more careful diagnosis, would have found their places in other columns. The

'Cholera Morbus,' too, has been necessarily blended in the tables with the 'Epidemic Cholera;' for, notwithstanding every precaution, it was found that these forms of disease could not be accurately distinguished in most of the returns: whether, under this uncertainty, that form of the disease has increased in latter years in the ratio of increase exhibited in 1816 and 1817, or not, it is difficult to judge; but there have at least occurred, on several occasions, a kind of cholera, very frequent in its attacks, and in all respects answering the definition of Cullen.

"To ascertain, therefore, the true proportional mortality of the prevailing epidemic cholera, recourse may be had to those formidable visitations in particular corps, which form part of the subject of the preceding narrative, and the result of this inquiry gives 767 cases, and 211 deaths, amongst the European troops; and 4065 cases, with 1544 deaths, amongst the natives.* We have thus the proportion of twenty-seven and a half per centum in the former, and nearly thirty-eight in the latter, which, considering that this disease runs its fatal course very generally within twelve hours, sufficiently marks it as one of the most formidable that has ever afflicted the human race."

*Fort St. George; 31st December*, 1822.

The epidemic has continued to hover about India up to the present time, but it would swell this work to too great a size to attempt more than a faint history of its first visitations in the numerous countries it has passed over; and we must therefore leave India, to examine how it penetrated to the south and east, into all the islands of the Indian seas, and into China; and how it spread to the west, over Persia, Arabia, Syria, and Russia.

It appeared in the small island of Penang, or Prince of Wales's island, about the 23d October, 1819, and carried off 800 persons in the course of a few months; it visited Queda, on the opposite coast, and Malacca, in the month following; and reached Acheen, on the coast of Sumatra, in the end of December. It continued to prevail in many of these islands during the year 1820; and, in the autumn, the government of Manilla, conceiving it to be contagious, ordered "that all

* This is evidently a most incorrect method of estimating the general mortality of the epidemic; for it is, in fact, only selecting the worst description of cases; and I would not even reject the cases of cholera morbus, in calculating the proportionate mortality, since it is notorious that this disease was generally a precursor of the other, and degenerated into it, if not checked by medicine. I believe, therefore, that the preceding table may be considered as exhibiting an accurate statement of the mortality from the epidemic in the army of Madras.

ships reaching Manilla from Penang, Malacca, Singapore, and Batavia, should perform quarantine for forty days outside the harbour." But, in spite of this precaution, it broke out there between the 1st and 3d of October, immediately after a most violent and destructive storm, and continued to rage in the island with great fury for some months. About the middle of this year, it committed great ravages in Siam; and, towards the end of it, it broke out in Canton, Whampoa, and Macao, where the alarm produced by it among the Chinese merchants was so great as to occasion a serious interruption to commerce. In the spring of 1821 it visited Batavia and other towns in the island of Java, and about the end of this year it spread into the countries of Cochin China and Jung King. It has reappeared in many of these countries, since the period under consideration, and probably few places in the Indian Archipelago have entirely escaped; but our scanty information from these distant regions will not enable us to enter more fully into this part of the history of the epidemic.

(To be continued.)

---

## Figures and Tables

**Figure f1:**